# Early warm-season mesoscale convective systems dominate soil moisture–precipitation feedback for summer rainfall in central United States

**DOI:** 10.1073/pnas.2105260118

**Published:** 2021-10-18

**Authors:** Huancui Hu, L. Ruby Leung, Zhe Feng

**Affiliations:** ^a^Atmospheric Sciences and Global Change Division, Pacific Northwest National Laboratory, Richland, WA 99352

**Keywords:** mesoscale convective systems, soil moisture–precipitation feedbacks, land–atmosphere coupling, hydrologic footprints, central US

## Abstract

Land–atmosphere interactions play an important role in summer rainfall in the central United States, where mesoscale convective systems (MCSs) contribute to 30 to 70% of warm-season precipitation. Previous studies of soil moisture–precipitation feedbacks focused on the total precipitation, confounding the distinct roles of rainfall from different convective storm types. Here, we investigate the soil moisture–precipitation feedbacks associated with MCS and non-MCS rainfall and their surface hydrological footprints using a unique combination of these rainfall events in observations and land surface simulations with numerical tracers to quantify soil moisture sourced from MCS and non-MCS rainfall. We find that early warm-season (April to June) MCS rainfall, which is characterized by higher intensity and larger area per storm, produces coherent mesoscale spatial heterogeneity in soil moisture that is important for initiating summer (July) afternoon rainfall dominated by non-MCS events. On the other hand, soil moisture sourced from both early warm-season MCS and non-MCS rainfall contributes to lower-level atmospheric moistening favorable for upscale growth of MCSs at night. However, soil moisture sourced from MCS rainfall contributes to July MCS rainfall with a longer lead time because with higher intensity, MCS rainfall percolates into deeper soil that has a longer memory. Therefore, early warm-season MCS rainfall dominates soil moisture–precipitation feedback. This motivates future studies to examine the contribution of early warm-season MCS rainfall and associated soil moisture anomalies to predictability of summer rainfall in the major agricultural region of the central United States and other continental regions frequented by MCSs.

## Significance

Soil moisture can significantly influence precipitation through soil moisture–precipitation feedback. Previous studies of soil moisture–precipitation feedback focused on the total precipitation, confounding the distinct roles of different storm types. Mesoscale convective systems (MCSs) are the largest form of deep convective storms, contributing 30 to 70% of warm-season rainfall in the central United States. Using unique datasets of MCS and non-MCS rains and soil moisture sourced from these rains, analyses revealed the dominant role of early warm-season (April to June) MCS rainfall in summer (July) MCS and non-MCS rainfall through positive and negative soil moisture–precipitation feedback, respectively. These results underscore the importance of understanding and modeling MCSs in the significant grain growing region of the central United States.

Soil moisture can affect precipitation through many pathways. Wetter-than-normal soils can effectively increase the moist static energy in the planetary boundary layer (PBL) and favor moist convection (1–3). On the other hand, drier-than-surrounding soils can induce mesoscale circulations that converge moisture toward the drier locations and initiate convection (4–8). While the latter mechanism tends to homogenize the spatial variability of soil moisture by inducing rainfall over dry soils, the former pathway can result in persistent wet/dry soil moisture states important at larger spatial scales and longer timescales (9, 10). These contrasting modulations of the spatiotemporal variability of soil moisture by precipitation can affect subsequent rainfall differently through land–atmosphere interactions.

Understanding soil moisture–precipitation feedback is important for improving understanding of subseasonal-to-seasonal predictability of precipitation in regions with strong land–atmosphere interactions (11–13). Located in a transitional zone between the arid, western United States and the humid, eastern United States, the central United States is a hotspot of coupling between soil moisture and precipitation in summer when evapotranspiration (ET) responds strongly to soil water availability and affects subsequent PBL development and convection (14–17). Under favorable thermodynamic and dynamical environments, afternoon convection can grow into mesoscale convective systems (MCSs) spanning >100 km and persisting much longer than isolated convection (18). These longer-lasting, propagating systems account for 30 to 70% of warm-season rainfall and over 50% of extreme rainfall in the central United States (19–22).

Compared with non-MCS rainfall, which consists mainly of rainfall from isolated deep convection and stratiform rain, MCS rainfall is on average ∼7 times stronger in rain intensity, although it occurs less frequently in space and time (23). Therefore, MCS and non-MCS rainfall have distinct footprints on land surface processes (24). In a warming climate that modulates the atmospheric and terrestrial environments, MCS and non-MCS rainfall may respond differently (25, 26) and manifest different changes in land–atmosphere interactions due to changes in the background conditions (27, 28). The potentially contrasting interactions of MCS and non-MCS rainfall with the changing environments may have implications for water availability in the major agricultural region of the central United States, motivating the need to understand their individual roles in soil moisture–precipitation feedback. Notably, previous land–atmosphere interaction studies mainly consider the total rainfall (9, 10, 29), despite the distinctive spatiotemporal characteristics of its MCS and non-MCS components (23) that may confound soil moisture–precipitation feedback. This study provides evidence that MCS and non-MCS rainfall in the early warm season (April to June) can induce soil moisture anomalies at different space and time scales, distinguishing their impacts on summer (July) MCS and non-MCS rainfall.

To reveal the roles of MCS and non-MCS rainfall in soil moisture–precipitation feedback in the central United States, we use a high-resolution (1/8° and hourly) database that associates the observed rainfall at each hour and grid location with MCS versus non-MCS rain events using an MCS tracking algorithm (24, 25, 30). This MCS dataset is combined with a soil moisture dataset derived from high-resolution (4 km) land surface model simulations with numerical tracers to “tag” MCS and non-MCS rainfall separately and track their partitioning into ET, soil moisture storage, and runoff (24). This dataset allows the total soil moisture to be partitioned into soil moisture components sourced from antecedent MCS or non-MCS rainfall. More details on the development of these datasets over the United States are provided in the *Materials and Methods* section. With these datasets, we spatially determine whether rain occurs preferentially over wetter/drier-than-surrounding soils and temporally determine whether rain occurs preferentially over wetter/drier-than-usual periods. More specifically, the spatial and temporal preferences of soil moisture anomalies for July afternoon (1500 to 2100 local time) and nighttime (2100 to 0300 local time) rainfall are analyzed using different methods (9, 10) to quantify the percentile values of spatial and temporal soil moisture anomalies associated with MCS and non-MCS rainfall events at the subdaily scale. In addition, the lagged effect of soil moisture on ET and rainfall in subsequent pentads is analyzed using a set of land–atmosphere coupling indices (13). The statistical significance of the results is tested against a null hypothesis of no land-driven feedback. The baseline for the null hypothesis is determined using simulation experiments produced by an analytical model of soil moisture responding to randomized rainfall time series (reference *SI Appendix*, *Supplementary Information Text* for more details).

## Soil Moisture Preference for MCS and non-MCS Rain at Subdaily Timescale

**Fig. 1.**
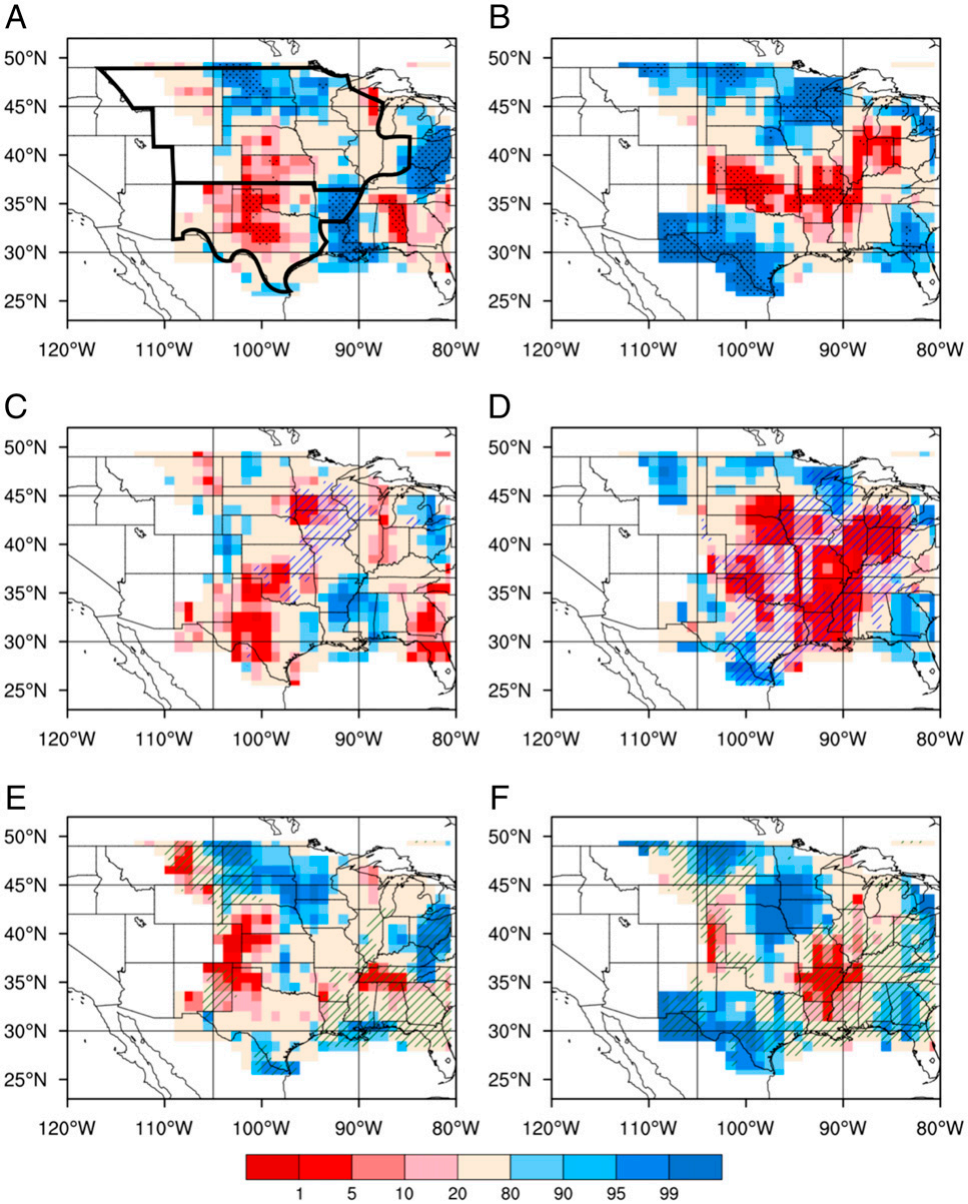
Spatial and temporal preference of soil moisture for afternoon non-MCS rainfall. Spatial preference (*Left*) and temporal preference (*Right*) of total soil moisture anomalies for afternoon non-MCS rainfall (*A* and *B*) and soil moisture anomalies contributed by antecedent MCS rainfall (*C* and *D*) and antecedent non-MCS rainfall (*E* and *F*). Soil moisture preferences are indicated by the percentile values of anomalies in soil moisture spatial gradients (*Left*) and anomalies of soil moisture (*Right*) for each 5° by 5° box tested against null distributions using bootstrap sampling. The statistical significance of percentile values (color shading) <5 or >95 is further tested against a null distribution with land-driven feedbacks removed. Stippling in *A* and *B* indicates areas with significant dry/wet preferences exceeding the 95% confidence level from the additional test. Blue (green) hatching in *C*–*F* indicates areas where soil moisture preferences (*A* and *B*) strongly correlate with the soil moisture sourced from MCS (non-MCS) rain. See more details in *Materials and Methods* and *SI Appendix*, *Supplementary Information Text* for determination of the spatial and temporal preference and the tests of significance. The thick black lines in *A* delineate the NGP and SGP, which comprise the central US region.

Both MCS and non-MCS rainfall frequently occur east of the Rocky Mountains (*SI Appendix*, Fig. S1), so we focus on the central United States in our analysis. Afternoon non-MCS rainfall, which accounts for 75% of the total afternoon rainfall, preferentially occurs over drier-than-surrounding (Fig. 1*A*) and drier-than-usual (Fig. 1*B*) soils in large areas over the central United States (stippled red areas, *P* < 0.05). The spatial preference is consistent with previous studies based on the total amount of afternoon rainfall (9, 10, 31), underscoring the importance of thermal circulation induced by soil moisture gradients in triggering afternoon deep convection over drier soils. The predominant drier-than-usual temporal preference for non-MCS rainfall in the Southern Great Plains (SGP) is also consistent with a previous study based on the total afternoon rainfall (10). In the SGP, where the atmosphere is sufficiently moist during daytime because of a residual of abundant moisture transported by the nocturnal Great Plains Low-Level Jet (GPLLJ) (25, 32, 33), larger-than-usual sensible heat flux over drier soils is a dominant factor for convection initiation. The latter is also supported by an analysis of triggering feedback strengths (*SI Appendix*, Fig. S2 *A* and *B*). In contrast, with the limited poleward extent of the GPLLJ, the atmosphere in the Northern Great Plains (NGP) is less thermodynamically favorable for convection. With stronger westerly background winds in the NGP compared to the SGP, rainfall may preferentially occur over wetter soils (Fig. 1*B*), as convection initiated over dry soils may be advected to wet soil patches that strengthen convection and provide moistening to increase the likelihood of rainfall generation (27).

Soil moisture anomalies in July are contributed by soil moisture anomalies on April 1 (when tagging of precipitation begins) and early warm-season (April to June) rainfall. The latter is of particular interest given our goal to better understand soil moisture–precipitation feedback at the subseasonal timescale. Based on the tracer-enabled land surface model simulations, more than 40% of the July 1 soil moisture in the top first meter comes from early warm-season rainfall tagged by the tracers (*SI Appendix*, Fig. S3 *C*–*F*). With the soil moisture dataset, the spatial and temporal preference of soil moisture for afternoon non-MCS rainfall is further analyzed for soil moisture sourced from early warm-season MCS and non-MCS rainfall separately (Fig. 1 *C*–*F*).

When comparing the spatial patterns of soil moisture anomalies, the drier-than-surrounding soil moisture anomalies sourced from MCS rainfall in the SGP (Fig. 1*C*) contribute more to the total soil moisture anomalies (Fig. 1*A*) favoring afternoon non-MCS rainfall, while the soil moisture anomalies sourced from non-MCS rainfall is shifted slightly northwestward (Fig. 1*E*) compared to the total soil moisture anomalies. This difference can be attributed to the higher intensity and larger area of MCS rainfall (23), which can effectively produce coherent mesoscale soil moisture anomalies with substantial spatial scale and strength. MCS rainfall can also infiltrate deeper into the soil that helps to maintain the soil moisture anomalies over longer periods. In contrast, non-MCS rain with smaller spatial scale and amount is less likely to generate coherent mesoscale patches of soil moisture anomalies large enough to induce thermal circulation and convection (*SI Appendix*, Fig. S3 *A*, *B*, and *G*). In the SGP, the drier-than-usual soil moisture anomalies (Fig. 1*B*) are also dominated by those sourced from MCS rainfall (Fig. 1*D*). However, soil moisture anomalies sourced from non-MCS rainfall (Fig. 1*F*) play a role in the wetter-than-usual soil anomalies in the NGP where atmospheric moistening is important for afternoon convection.

Similar analysis for afternoon MCS rainfall, which accounts for 25% of the total afternoon rainfall in July, shows much weaker drier-than-surrounding soil moisture anomalies but substantial wetter-than-usual soil moisture preferences (*SI Appendix*, Figs. S2 *C* and *D* and S4). Notably, a large fraction of MCS events in the central United States are initiated upwind near the Rocky Mountains Foothills. Subsequent convective aggregation generates MCSs that propagate eastward to the central United States (22), so drier-than-surrounding soil moisture anomalies may not be necessary for initiating MCSs in the central United States. Instead, soil moisture in the central United States may contribute to moistening of the PBL and favor downwind and upscale growth of convection into convection with larger, more organized structure, and enhance MCS rainfall amount. The contrasting temporal preferences between afternoon non-MCS and MCS rainfall emphasize the important role of surface moistening in supporting convective aggregation of MCSs versus the role of drier soils in convective triggering of non-MCS rain, as hinted by other studies (34, 35). Decomposing the wetter-than-usual soil conditions into components sourced from preceding MCS and non-MCS rainfall reveals the dominant source from early warm-season MCS afternoon rain (*SI Appendix*, Fig. S4 *B*, *D*, and *F*).

**Fig. 2.**
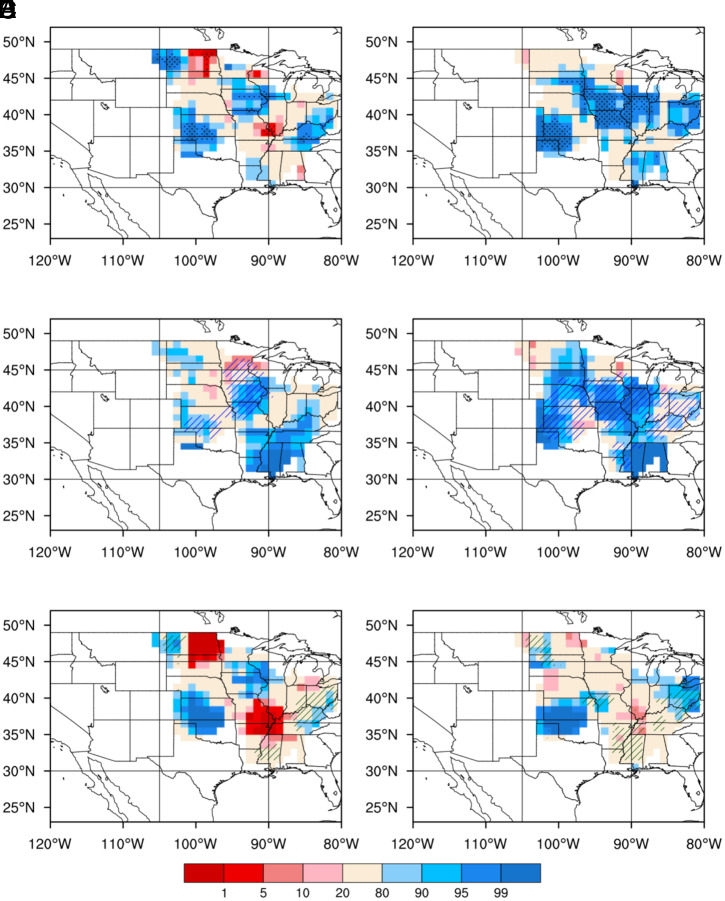
Spatial and temporal preference of soil moisture for nighttime MCS rainfall. Spatial preference (*Left*) and temporal preference (*Right*) of total soil moisture anomalies for nighttime MCS rainfall (*A* and *B*) and soil moisture anomalies contributed by antecedent MCS rainfall (*C* and *D*) and antecedent non-MCS rainfall (*E* and *F*). See more details for explanations of the color shading and stippling/hatching in the caption of Fig. 1.

In total, 49% of nighttime rain is contributed by MCS rainfall, which preferentially falls over wet soils in the central United States (Fig. 2 *A* and *B*). The preferred wet soil moisture conditions for nighttime rainfall have also been noted previously (9). As soil moisture gradient induced mesoscale circulation is damped by the increased stability of the PBL at night, low-level moisture becomes a critical element to sustain MCS rainfall (36, 37). The wet soil anomalies that favor MCS rainfall during nighttime are attributed to soil moisture anomalies sourced from both antecedent MCS and non-MCS rainfall (Fig. 2 *C*–*F*). However, the pattern of total soil moisture preferences in the Central Great Plains (Fig. 2 *A* and *B*) are more strongly correlated with the pattern of MCS contributed soil moisture (hatched areas in Fig. 2 *C* and *D*). The consistent temporal preferences for both afternoon and nighttime MCS rainfall over wet soils (Fig. 2 and *SI Appendix*, Fig. S4) imply areas that receive more early warm-season MCS rainfall may also receive more MCS rainfall in July. Hence, the moistening effect of MCS- and non-MCS–sourced soil moisture on MCS rainfall is further examined through analysis of land–atmosphere coupling strengths.

## Soil Moisture–Precipitation Feedback at Subseasonal Timescale

While the thermal modulation of PBL through sensible heat flux induced by dry soil moisture anomalies occurs more instantaneously at a subdaily timescale, the moistening of the PBL through ET is more gradual and accumulative. Increased soil moisture due to a single rainfall event can effectively increase the humidity within the PBL for several days over a larger area through advection and mixing (38). To quantify the role of soil moisture on longer-term MCS rainfall through ET, we aggregate the July ET and rainfall into six pentads and calculate two coupling indices of the “two legged” processes of soil moisture–precipitation feedback: a terrestrial leg quantified by the lag correlation between initial soil moisture anomalies and ET in the following pentad, and an atmospheric leg quantified by the lag correlation between pentad ET and the 5-d lagged pentad rainfall (see Eqs. **1**–**3** in *Materials and Methods*). These two coupling indices link the land surface states to the atmospheric responses (13, 15) and emphasize the causal relationship between initial soil moisture and rainfall in the subsequent period through ET.

**Fig. 3.**
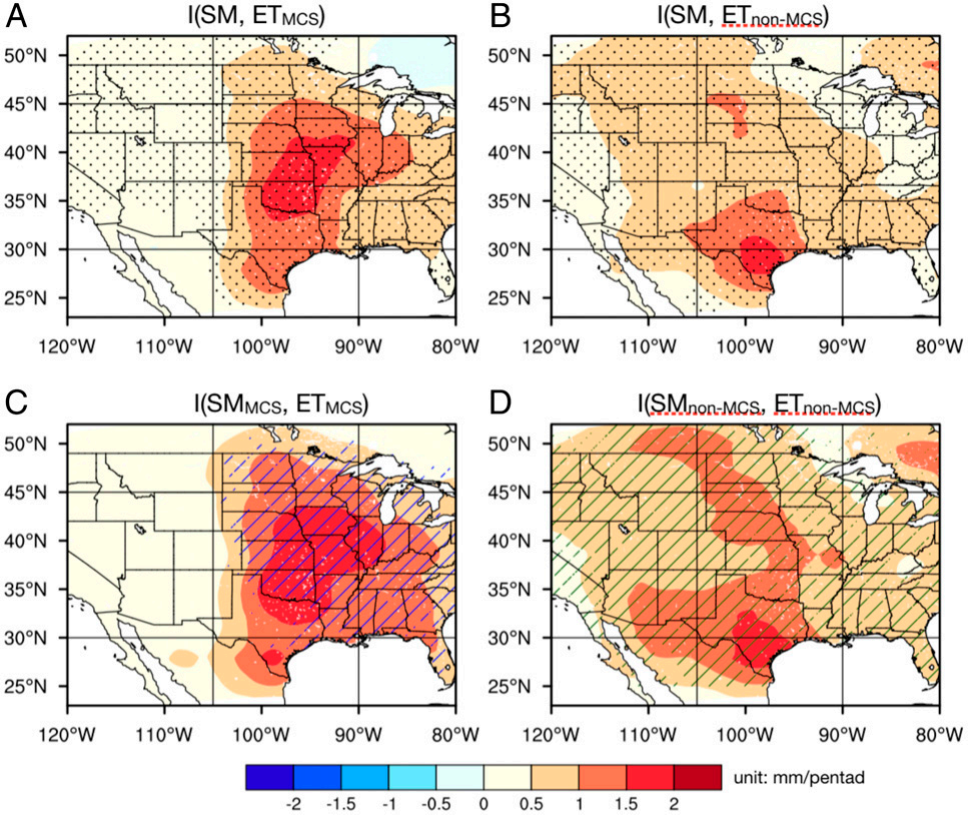
Coupling strengths between 1-m soil moisture and the subsequent pentad ET. Spatial distribution of the coupling strengths between 1-m soil moisture and the subsequent pentad ET of the MCS (*Left*) and non-MCS (*Right*) components. Stippling in *A* and *B* indicates areas with significant correlation (ρ > 0.3, *P* < 0.05) between the two variables. All areas in *C* and *D* are significantly correlated, hence not stippled. The blue (green) hatched areas in *C* (*D*) indicate where MCS (non-MCS) contributed soil moisture has significantly stronger (weaker) coupling with ET_MCS_ than ET_non-MCS_.

Pentad ET components sourced from preceding MCS and non-MCS rainfall are both strongly coupled with the initial soil wetness in the central United States (Eq. **1** and Fig. 3). This is expected in July; when there is an abundant energy supply, latent heat fluxes are mainly limited by water availability. The maximum coupling strength between the MCS-sourced soil moisture and ET collocates noticeably with the early warm-season MCS rainfall (Fig. 3*C* and *SI Appendix*, Fig. S3*A*) and is consistent with the role of antecedent MCS rainfall in these surface water components sourced from MCSs. In contrast, the maximum coupling strength between the non-MCS–sourced soil moisture and ET maximizes along a meridional band ∼100°W (Fig. 3*D*); this is indicative of a stronger local sensitivity of non-MCS ET to soil moisture than to rainfall variations (39). While ET sourced from MCS and non-MCS rainfall both manifest significant correlations with the total soil moisture (95% confidence level), each ET component is more significantly correlated with its source soil moisture component (hatched areas in Fig. 3 *C* and *D*). Although this is mainly by design of the water tagging, it confirms that cross-component correlations (i.e., SM_MCS_ and ET_non-MCS_, SM_non-MCS_ and ET_MCS_) due to other pathways (e.g., synchronized dynamics of these components) are limited, thus emphasizing the causal relationships between soil moisture and ET for each rainfall component.

**Fig. 4.**
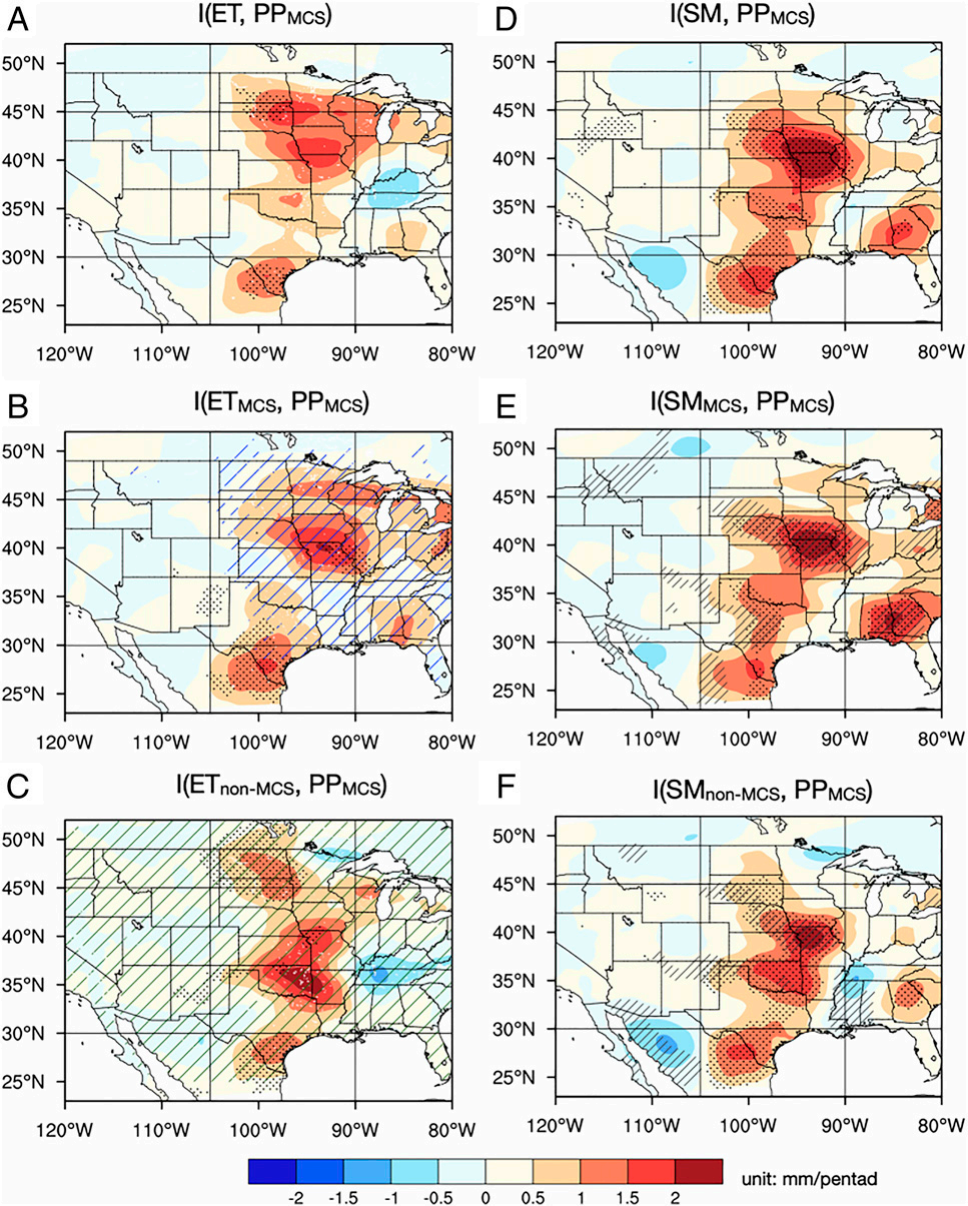
Coupling strengths between initial soil moisture and subsequent July pentad rainfall and the atmospheric leg of the coupling process chain in July. (*Left*) Coupling strengths between pentad ET and 5-d lagged pentad MCS rainfall (PP_MCS_), representing the atmospheric leg connecting soil moisture with subsequent rainfall through total ET (*A*) and ET sourced from MCS rain (*B*) and non-MCS rain (*C*). (*Right*) Coupling strengths between initial soil moisture and pentad MCS rainfall (PP_MCS_) for the total soil moisture (*D*) and soil moisture sourced from MCS rain (*E*) and non-MCS rain (*F*). The coupling strengths have units of mm/pentad. On the *Left*, stippling indicates areas with significant correlation (ρ > 0.3, *P* < 0.05) between the two variables. The blue (green) hatching in *C* (*E*) indicate areas where ET sourced from antecedent MCS (non-MCS) rainfall has significantly stronger (weaker) coupling with MCS rainfall than non-MCS rainfall. On the *Right*, stippling indicates areas with significant coupling strengths (exceeding 95% confidence level) tested against a null distribution with land-driven feedbacks removed; areas where positive coupling is closely related to ET are hatched in *D* and *F* (i.e., areas where the direct correlation between soil moisture and 5-d lagged pentad rainfall is statistically significant and larger than the partial correlation with the effect of ET removed, reference *SI Appendix*, Fig. S7 *A*–*D*). Pentad rainfall (PP) and soil moisture (SM) data are smoothed with an ∼5-degree filter prior to computing the coupling strengths. Soil moisture is at 1-m depth.

The atmospheric leg quantified by the lagged response of rainfall to ET (Eq. **2**) also shows a strong coupling between pentad ET and MCS rainfall in the following pentad in the Central Great Plains (Fig. 4*A*), confirming the important role of moisture supply to MCS rainfall noted in the analysis of the soil moisture preference of MCS rain (Fig. 2). Such a strong coupling for MCS rainfall is contributed by both the MCS and non-MCS ET components (Fig. 4 *B* and *C*). Positive coupling in the atmospheric leg can be contributed by the response of PBL dynamics to surface fluxes, which then affect clouds and precipitation (15, 40). Rainfall persistence is unlikely to contribute to the lag correlation between pentad ET (influenced by coincident rainfall) and rainfall in the following pentad because the autocorrelation of July MCS rainfall diminishes quickly with an e-folding time of ∼1 d (*SI Appendix*, Fig. S5), and there is no statistically significant autocorrelation of 5-d lagged pentad MCS rainfall (*SI Appendix*, Fig. S6*B*) in areas of the central United States with strong coupling between ET and MCS rainfall (Fig. 4*A*). Importantly, autocorrelation of pentad MCS rainfall at 10-d lag is significant in those areas (*SI Appendix*, Fig. S6*E*). The difference between 5-d and 10-d lagged autocorrelations for MCS rainfall demonstrates a minimal role of rainfall persistence but a higher likelihood of land-driven feedback through ET (14) that operates at longer timescales. This is further supported by spectral analysis of MCS rainfall showing the absence of MCS rainfall variability at the frequency range of ∼10 d that could have contributed to the enhanced 10-d lagged pentad MCS autocorrelation (*SI Appendix*, Fig. S6*G*).

**Fig. 5.**
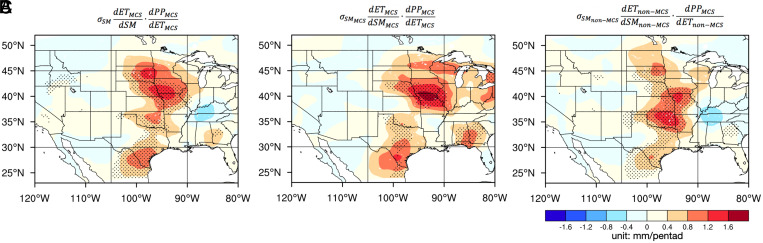
Two-legged coupling strengths between initial soil moisture and subsequent pentad rainfall in July. Product of the two-legged coupling indices (shown in Figs. 3 and 4 *A*, *B*, and *C*) normalized by the SD of ET, as shown by the equations on *Top* of each panel, to indicate the coupling between soil moisture and rainfall through the terrestrial and atmospheric legs for the total soil moisture (*A*) and soil moisture sourced from MCS (*B*) and non-MCS (*C*) rainfall. The stippled areas in the *Right* of Fig. 4 are also shown here for comparison.

Having demonstrated the connection between initial soil moisture and subsequent rainfall through ET using the two-legged coupling indices, we now examine the coupling strength between soil moisture and rainfall using lagged correlation (Eq. **3** in *Materials and Methods*). To emphasize the role of ET in the process chain connecting soil moisture and rainfall, we compare the lagged correlation between initial soil moisture and pentad rainfall (*Right* of Fig. 4) with a two-legged coupling metric (Fig. 5), defined as the product of the two coupling indices (41) of the terrestrial and atmospheric legs (Fig. 3 and *Left* of Fig. 4) to explicitly account for the role of ET in the soil moisture–precipitation coupling. The two metrics quantifying soil moisture–precipitation coupling (Fig. 4, *Right* and Fig. 5) show very similar spatial patterns with maximum coupling in the Central Great Plains. These patterns are dominated by the patterns of the atmospheric leg (Fig. 4, *Left*), confirming the role of ET in the process chain.

To further support the role of ET in the process chain, we compare the correlation between initial soil moisture and 5-d lagged pentad rainfall (*Right* of Fig. 4) and the partial correlation between these variables after their respective linear regressions with the pentad ET are removed (Eq. **4** in *Materials and Methods* and *SI Appendix*, Fig. S7). Consistent with the important role of ET in the soil moisture–precipitation relationship, the partial correlation with the effect of pentad ET removed shows larger values only in northern Texas (*SI Appendix*, Fig. S7) where ET and rainfall coupling is weak (Fig. 4*B*). Importantly, most of the areas where the partial correlation is weaker than the actual correlation (hatched in Fig. 4 *E* and *F*) collocate with areas in the central United States where the correlation between soil moisture and precipitation is significant (stippled areas in Fig. 4, *Right*), as tested against a null distribution with land-driven feedbacks removed (*P* < 0.05; see *Materials and Methods*). That is, removing the effect of pentad ET suppresses the significant correlation between soil moisture and pentad rainfall. Hence, the partial correlation analysis supports the important role of ET in connecting the initial soil moisture with the 5-d lagged pentad rainfall in areas with strong coupling strengths.

For MCS rainfall, the strong coupling between soil moisture and July rainfall over the central United States is supported by strong couplings between soil moisture and ET and between ET and precipitation, with ET sourced from both antecedent MCS and non-MCS rainfall (Fig. 3 and *Left* of Fig. 4). Consequently, soil moisture anomalies contributed by both antecedent MCS and non-MCS rainfall are important for enhancing July MCS rainfall (Fig. 4 *E* and *F*). This positive feedback is consistent with the nighttime MCS rainfall preferentially occurring over wetter-than-usual soils at subdaily timescale (Fig. 2).

**Fig. 6.**
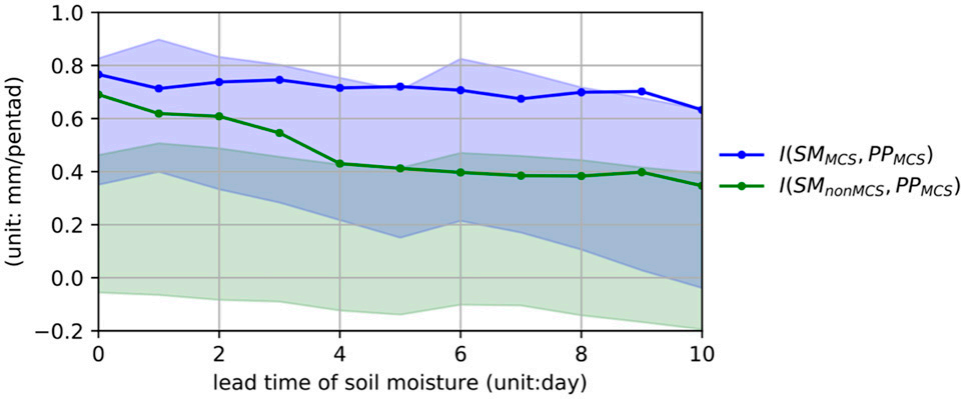
Soil moisture–precipitation coupling strengths as a function of lead time. Changes of coupling strengths (unit: mm/pentads) of initial soil moisture at 1 m with pentad MCS rainfall as a function of lead time of the initial soil moisture. Blue and green shading indicates the 5 to 95% ranges of coupling strengths from a null distribution with land-driven feedbacks removed. Results are averages over the central United States (31 to 49ºN, 105 to 90ºW).

Although MCS rainfall is strongly coupled to soil moisture contributed by both antecedent MCS and non-MCS rainfall (Fig. 4 *E* and *F*), the coupling manifests at different timescales (Fig. 6). More specifically, the coupling strength associated with non-MCS–sourced soil moisture decreases by 50% with lead time from 0 to 10 d, while the coupling strength associated with MCS-sourced soil moisture remains strong even at a lead time of 10 d. This indicates a shorter memory of soil moisture sourced from non-MCS rainfall compared to MCS rainfall. The difference in soil memory may be explained by the shallower infiltration of the lighter non-MCS rainfall into the soil and hence, faster turnover of the soil moisture anomalies through soil evaporation (24). In contrast, MCS rainfall marked by stronger intensity can infiltrate deeper into the soil to sustain the soil moisture anomalies and their impact on subsequent MCS rainfall through plant and soil hydraulics over longer durations.

## Discussion and Summary

**Fig. 7.**
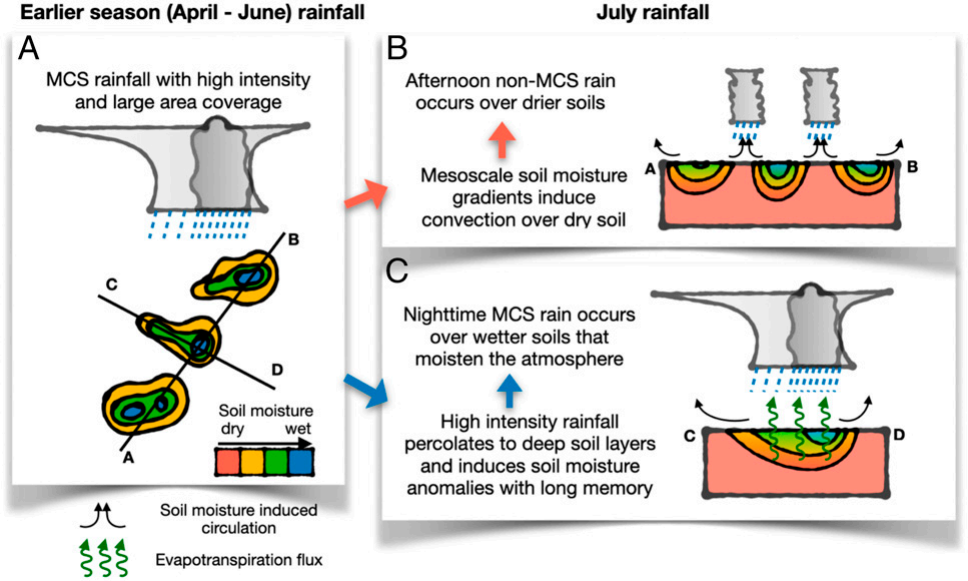
Schematic of the dominant effect of early warm-season MCS rainfall on summer rainfall in the central United States. Early warm-season MCS rainfall with high intensity and large areas (*A*) can induce mesoscale soil moisture gradients that favor non-MCS rainfall over dry soils at the subdaily timescale (*B*). On the other hand, early warm-season MCS rainfall can percolate to deep soils with long memory, moistening the atmosphere and favoring MCS enhancement over wet soils in summer (*C*).

In this study, we investigate the interactions between soil moisture and rainfall in the central United States from the unique perspective of how early warm-season (April to June) MCS and non-MCS rainfall affects July MCS and non-MCS rainfall through the distinct footprints of the two rainfall components on soil moisture. Besides differences in rain intensity and area, MCS and non-MCS rainfall also differ in their diurnal timing and the remote (Rocky Mountain Foothills) versus local (central United States) convective triggering, all of which play important roles in how MCS and non-MCS rainfall influence soil moisture–precipitation feedback. We find that MCS rainfall produces more spatially coherent soil moisture heterogeneity or mesoscale soil moisture gradient because of its larger intensity and area coverage compared to non-MCS rainfall (Fig. 7A). Hence, soil moisture anomalies associated with early warm-season MCS rainfall can more effectively induce mesoscale thermal circulation and triggering of afternoon convection, mostly associated with non-MCS rainfall (Fig. 7B). Early warm-season MCS rainfall therefore plays an important role in inducing rainfall over dry soils (i.e., negative soil moisture–precipitation feedback) associated with afternoon non-MCS rain. Note that the background zonal gradients in soil moisture and vegetation in the central United States featuring a large-scale, meridional, semiarid-to-humid transition can also contribute to soil moisture gradients and thus soil moisture–precipitation feedback. However, such effects do not play a role in our analysis as we focus on soil moisture anomalies with the seasonal cycle removed.

On the other hand, both early warm-season MCS and non-MCS rainfall provide a critical source of soil moisture that moistens the PBL and enhances July MCS rainfall, with a large fraction occurring during night hours. Such positive feedback between soil moisture and MCS rainfall extends beyond the subdaily scale, as evidenced by the strong positive coupling between soil moisture and MCS rainfall amount in the following pentad. However, soil moisture anomalies produced by antecedent non-MCS rainfall are confined closer to the surface due to the lighter rainfall intensity, so their impact on subsequent MCS and non-MCS rainfall tends to be shorter lived. This is in stark contrast to antecedent MCS rainfall that infiltrates deeper in the soil to produce a longer-lived soil moisture footprint and prolonged impact on summer rainfall (Fig. 7C). Overall, faster decay of the coupling between non-MCS–sourced soil moisture and MCS rain (Fig. 6) suggests that non-MCS rainfall in the early warm season plays a less important role in predicting summer rainfall in the central United States.

Our analysis provides evidence that wet soils favor MCS rainfall during the nighttime in the central United States. This is consistent with results from an idealized model where the maximum value of convective available potential energy for continental convective storms scales with available surface moisture (42). In the Sahel, a recent study (28) shows that dry soils may intensify MCSs by inducing multiscale processes that converge monsoonal moisture over the dry soils—which more than offsets the reduced ET from the local dry soils and produces more intense MCSs. In both the Central Great Plains and the Sahel, mature MCSs are not triggered locally (26, 43, 44) so soil moisture–precipitation feedback is not related to convective triggering of MCSs. Despite this similarity, the central United States receives abundant moisture transported by the GPLLJ, so local, dry soils are less likely to induce significant changes in moisture convergence as found in the drier Sahel where local, dry soils and associated warm temperature anomalies can enhance the monsoonal moisture convergence into the region. Studies using observations and numerical simulations will be useful to gain further insights into the role of soil moisture anomalies on MCSs in different regions with their unique, large-scale circulations and local land surface characteristics.

The soil moisture dataset used in this study is subject to uncertainties related to limitations of the land model in representing the total soil moisture and partitioning into the components sourced from antecedent MCS and non-MCS rainfall using water tracers (24). Our results may also be sensitive to the tracking of MCS rainfall (see more details in *Materials and Methods*), which can affect the partitioning of total rainfall into its MCS and non-MCS components. Similar to previous observational studies of land–atmosphere interactions, our results are based on statistical analysis of soil moisture and precipitation, which could be prone to misinterpretation. For example, rainfall persistence may contribute to the correlation between initial soil moisture and 5-d lagged MCS rainfall. We note also that unequivocally excluding the role of rainfall persistence is extremely challenging, if not impossible. Still, our various analyses support, if not prove, the idea that we are seeing the effects of feedback. For example, we find no significant autocorrelation of 5-d lagged pentad MCS rainfall in the central United States where soil moisture–precipitation coupling is significant. In contrast, the autocorrelation of 10-d lagged pentad MCS rainfall is significant in that region, lending support to the presence of soil moisture–precipitation feedback, contributing to the rainfall persistence at longer timescales (14, 45, 46). In sum, multiple lines of evidence including comparison of the lagged correlation between soil moisture and rainfall against a two-legged coupling metric and partial correlation with the effect of ET removed, significance tests against a null hypothesis of no land-driven feedbacks using randomized rainfall, and the short e-folding timescale of rainfall autocorrelation have enhanced confidence in the results and conclusions. Future studies comparing coupled land–atmosphere simulations with simulations in which soil moisture–precipitation feedback is disabled may be used to provide further support for the role of MCS rainfall in soil moisture–precipitation feedback (12, 39, 41).

This study has provided insights on land–atmosphere interactions by isolating the distinct land surface footprints of early warm-season MCS and non-MCS rainfall and their subsequent impacts on summer rainfall. Through its dominant influence on soil moisture in the deeper layer and at mesoscale, early warm-season MCS rainfall may influence the spatial distribution and amount of MCS and non-MCS rainfall in July and hence potentially provide a source of predictability for summer rainfall at subseasonal-to-seasonal timescales. However, demonstrating such predictability requires more extensive analysis and modeling experiments. Our results suggest that knowledge of the partitioning of early warm-season rainfall into MCS and non-MCS components, combined with monitoring of near-surface soil moisture, may provide useful constraints for modeling subsurface soil moisture with longer memory for improving prediction of July rainfall. Our results also underscore the importance of representing MCS in models used in weather and climate forecasting. Notably global climate simulations (45, 46) and regional simulations, including convection permitting simulations (26, 44), exhibit significant biases in MCSs in the central United States, particularly during summer. Model biases in simulating early warm-season MCS rain may disrupt the soil moisture–precipitation feedback loop for both MCS and non-MCS rainfall, limiting the ability of models to simulate summer rainfall in the major agricultural region of the central United States. Since MCSs occur frequently in many continental regions worldwide, our results motivate the need for more systematic investigations of the distinct roles of MCS and non-MCS rainfall in soil moisture–precipitation feedback.

## Materials and Methods

### Rainfall Dataset.

We use gridded precipitation data from the North American Land Data Assimilations System (NLDAS) (47) at 1/8° spatial resolution and hourly temporal resolution. NLDAS combines ground‐based rain gauges, radar, and satellite observations of precipitation over the United States. Precipitation in the warm season (April to August) is attributed to MCS using an MCS tracking algorithm (25). Summer precipitation in the central United States not produced by MCS is contributed by both isolated convection and nonconvective or stratiform precipitation and is collectively referred to as non-MCS rainfall. MCSs are identified if the major axis length of a precipitation feature (PF, defined as a contiguous rainy area with pixel‐level rainrate >1 millimeter hour^−1^) exceeds 200 km and persists for at least 4 h (see more details in ref. 25). The robustness of this MCS tracking method has been evaluated by comparing it with a more commonly used MCS tracking method based on satellite brightness temperature data and a more sophisticated MCS tracking method that uses the radar network in the United States (25, 44). Comparison of MCS rainfall characteristics and long-term trends from the MCS tracking using PF versus radar data shows consistency between the two (23). Here, we used data derived from the PF tracking method for 1997 to 2018 because the inclusion of radar and satellite data in the NLDAS precipitation estimates starting in 1997 improves the spatiotemporal distribution of hourly gridded rain gauge rainfall features (25). To understand the roles of MCS and non-MCS rainfall in soil moisture and the interactions with subsequent rainfall, the MCS and non-MCS components of precipitation are “tagged” separately in land surface model simulations. Note that the NLDAS precipitation data are interpolated to the 4-km-resolution grid of a water tracer enabled Noah-MP land surface model (WT-Noah-MP) to provide atmospheric forcing, and the resolution of precipitation and soil moisture is further adjusted by aggregation or smoothing for analysis of preferential soil moisture states and coupling indices (see more details for each analysis method described in *Analysis Methods*).

### Soil Moisture Data.

The soil moisture data used in this study is obtained from 22 y of simulations (1997 to 2018) using the Noah land surface model with multiple physics parameterizations (Noah-MP) (48). For each year, we perform two simulations covering March 1 to August 31, using atmospheric forcing from the NLDAS dataset (precipitation, radiation, near-surface air temperature, wind and humidity, and surface pressure). We use a similar domain as NLDAS but with a 4-km grid spacing to better represent land surface characteristics, such as soil and vegetation (using a 30-arc-s-resolution geographical dataset from https://www2.mmm.ucar.edu/wrf/users/download/get_sources_wps_geog.html). The NLDAS atmospheric forcing is interpolated to the land surface model domain at 4-km grid spacing as model input.

The land surface simulations use a unique water tracer–enabled version of Noah-MP (WT-Noah-MP) (30) to “tag” rainfall associated with MCS and non-MCS events, respectively, and track their subsequent transit in the soil. In each simulation, rainfall due to MCS or non-MCS events (only one type is tagged in each simulation) occurring in April to August is cumulatively “tagged” by water tracers. The “tagged” water can transit through different storages in the terrestrial system until it leaves the system as runoff or ET. The water tracer transit is represented by an additional set of equations that describe the dynamics of the partial storages and fluxes of the water tracers. Within each storage (e.g., canopy interception storage, each snow layer, each soil layer), the tracer input is assumed to be well mixed with the remaining storage so the tracer flux from each storage is partitioned from the total flux in proportion to the tracer mixing ratio (30). This water tracer–enabled version has been used to reveal the different roles of MCS and non-MCS rainfall on the surface water balance due to their differences in intensity, diurnal timing, and seasonal and spatial distributions (24). Particularly relevant are the differences in soil moisture profiles: MCS rainfall with greater intensity can produce higher pressure head and higher hydrologic connectivity and thus drive deeper percolation into the soils, while non-MCS rainfall with weaker intensity tends to stay in the topsoil layers (*SI Appendix*, Fig. S3 *H* and *I*). Such differences in soil moisture profiles can cause different responses to ET and different residence times in the soil that can affect soil moisture–precipitation feedback. The water tracer–enabled simulations are used in this study to understand the soil moisture–precipitation feedback associated with soil moisture derived from MCS or non-MCS rainfall.

In using the offline land surface simulations and observed precipitation for analysis of soil moisture–precipitation feedback, it is assumed that the observed precipitation carries in it the signal of any actual soil moisture–precipitation feedbacks and the land model driven by the observationally based precipitation from NLDAS retains the soil moisture dynamics influenced by the feedbacks. Our observation-driven analysis has some advantages compared to using coupled land–atmosphere simulations that are subject to model errors and uncertainties in producing precipitation related to parameterizations of atmospheric processes (e.g., convection, PBL, microphysics).

### Analysis Methods.

Our analysis primarily focuses on July, when the strongest interactions between soil moisture and rainfall are expected in the central United States (14, 49). We examine the role of soil moisture in afternoon and nighttime rainfall. We also integrate rainfall into ∼weekly scale to better understand the longer-term impact of soil moisture, with particular attention to the modulation of early warm-season (April to June) MCS and non-MCS rainfall on soil moisture. The moisture is “tagged” by rainfall type in April, because of its role in inducing MCS and non-MCS rainfall in July.

#### Spatial and temporal preference for afternoon and nighttime rainfall.

We analyze the effect of soil wetness on afternoon and nighttime rainfall following the method used by refs. 9 and 10. The rainfall (interpolated from the 1/8° NLDAS grid to the 4-km land model grid as the forcing data) and soil moisture from WT-Noah-MP on the 4-km grid are both aggregated to a 24-km grid to focus on mesoscale land–atmosphere interactions. Afternoon rainfall is defined as accumulated rainfall during 1500 to 2100 local time and soil moisture conditions that contribute to these afternoon rainfall events (6 h rainfall > 3 mm) are quantified by the pre-event soil moisture anomalies at 0900 local time after removing the seasonal cycle. Two variables on the 24-km grid are used to characterize the pre-event soil moisture anomalies. For the soil moisture spatial preference analysis, soil moisture spatial gradient is calculated as the difference of soil moisture anomalies (ΔSM’) between the location with maximum rainfall (L_max_) and the location(s) with minimum rainfall (L_min_) within a five grid point by five grid point (120 × 120 km) box centered at L_max_. For the soil moisture temporal preference analysis, the soil moisture anomaly at L_max_ (SM’(L_max_)) is used to represent the wetness relative to its own seasonal cycle. Rainfall events at grid cells with terrain (topographic height within a 120 × 120 km box > 300 m) and water body features (water bodies cover >5% of the area within a 120 × 120 km box) are excluded from the analysis as their influence on precipitation may confound interpretation of soil moisture–precipitation feedback.

The difference between the pre-event soil moisture conditions (ΔSM’ and SM’(L_max_)) and the soil moisture conditions of a control sample of days in nonevent years is compared against typical values to indicate its significance. To achieve adequate sample sizes, we pool together events and their corresponding control samples belonging to a fixed 5° × 5° box with their climatology removed to ensure comparability for different locations. For the 5° × 5° boxes with more than 25 events, the actual soil moisture anomaly difference between the events and the control samples $\bar{\Delta SM{'}_{e}}-\bar{\Delta SM{'}_{c}}$ or $\bar{SM{\left(L_{\max}\right)}_{e}}-\;\bar{SM{\left(L_{\max}\right)}_{c}}$ (where *e* represents events and *c* represents control samples) are compared with typical values of the differences by pooling both events and control samples together in a 1,000 bootstrapping sampling of a size equal to the events. The percentile of the null distribution (represented by the 1,000 bootstrapping tests) against which the actual differences are compared quantifies the significance of the spatial/temporal soil preferences for rainfall events. We use soil moisture in the top 1 m to account for the root zone. While refs. 9 and 10 focused on afternoon rainfall, we also perform analysis for nighttime rainfall (2100 to 0300 local time) as a large fraction of central US MCS rainfall occurs at night. Rainfall events occurring in the afternoon or nighttime are attributed to MCS or non-MCS events based on which type of events account for the majority (>50%) of the accumulated rainfall. To better present the spatial details of the spatial and temporal preferences, we show the percentile values at each 1° × 1° grid box representing the results of the 5° × 5° box centered at the grid.

The statistical significance of the spatial and temporal soil preferences (represented by percentile values) for rainfall events is further tested against a null hypothesis of no land-driven feedbacks. This null distribution is determined by using typical percentile values from 500 experiments produced by an analytical model representing soil moisture as a first-order Markov process. The analytical model is driven by permutation resampling of rainfall time series (see more details of the analytical model in *SI Appendix*, *Supplementary Information Text*). By randomizing the rainfall time series, any land-driven feedbacks to rainfall are removed while the soil memory of preceding rainfall is retained. The 500 experiments provide the baseline for the null hypothesis against which the actual soil preferences are tested.

Attributing the soil moisture preferences to soil moisture sourced from early warm-season (April to June) MCS and non-MCS rainfall is our primary interest. Therefore, we compute the soil moisture preferences for the same rainfall events using MCS- and non-MCS–contributed soil moisture components obtained from the WT-Noah-MP simulations described in *Soil Moisture Data*. Similar significance tests against the typical percentile values from the 500 experiments described above have been performed to confirm the significance of the dry (<5%) or wet (>95%) preferences (not shown). To emphasize the role of MCS- or non-MCS–sourced soil moisture on the soil preferences, hatching is used to indicate areas where soil moisture sourced from MCS/non-MCS rain is significantly correlated with the soil preference, tested against the 500 baseline experiments with land-driven feedbacks removed (hatched areas in Figs. 1 C*–*F and 2 C*–*F).

#### Coupling between soil moisture and pentad rainfall.

The effects of soil moisture on rainfall are evaluated based on pentad rainfall to understand the roles of soil moisture on longer time scales. To quantify the variation of pentad rainfall in association with soil moisture through its influence on surface energy fluxes, we use the “two legged” coupling metric (13, 15) to examine the possible linkages between soil moisture and rainfall in the terrestrial and atmospheric legs, each defined by a coupling index:

[1] $$\text{Terrestrial}\;\text{coupling}\;\text{index}:\;I\left(SM,\;ET\right)=\sigma_{SM}\frac{\mathrm{dET}}{\mathrm{dSM}}=\sigma_{ET}r\left(SM,ET\right),$$

[2] $$\text{Atmospheric}\;\text{coupling}\;\text{index}:\;I\left(ET,\;PP\right)=\sigma_{ET}\frac{\mathrm{dPP}}{\mathrm{dET}}=\sigma_{PP}\text{r}\left(ET,PP\right),$$

where *SM* is soil moisture at the beginning of each pentad in July, *ET* is pentad evapotranspiration, and *PP* is one pentad (5-d) lagged precipitation, σ is the SD, dET/dSM and dPP/dET are the linear regression slopes of ET on SM and PP on ET, and r is the correlation coefficient. The coupling indices are calculated for each 4-km grid cell from the WT-Noah-MP simulations after an ∼5-degree smoothing filter is applied. Note that we simplify the atmospheric leg as the coupling between ET and PP due to the lack of atmospheric variables (e.g., PBL height, near-surface moist static energy) from coupled simulations that are typically used in model-based analysis. In considering the diagnostic, the reader should keep in mind the possibility that non–land-related memory in precipitation processes, combined with passive responses of soil moisture and ET to this memory, may artificially inflate the diagnostic’s value.

The linkage between soil moisture and July rainfall is then examined by calculating their coupling strength using Eq. **3**:

[3] $$I\left(SM,\;PP\right)=\sigma_{SM}\frac{\mathrm{dPP}}{\mathrm{dSM}}=\sigma_{PP}r\left(SM,PP\right),$$

where the symbols in Eq. **3** are the same as those in Eq. **1**.

The soil moisture–precipitation feedback strength quantified by Eq. **3** is also compared with the product of Eqs. **1** and **2**, normalized by the SD of ET ($\sigma_{SM}\frac{\mathrm{dET}}{\mathrm{dSM}}\cdot \frac{\mathrm{dPP}}{\mathrm{dET}}$) to validate their coupling through the terrestrial and atmospheric pathways (see similar metric in ref. 41). Meanwhile, the role of ET is also examined by calculating the partial correlation (40), ρ[(SM, PP)⋅ET], between initial soil moisture and 5-d lagged rainfall with the effect of pentad ET removed, as defined by Eq. **4**.

[4] $$\text{Partial}\;\text{correlation}:\;\rho \left(SM,PP\right)\cdot ET=r\left(e_{SM,ET},\;e_{PP,ET}\right),$$

where ρ(SM,PP)⋅ET is the correlation between the residuals of initial SM $\left(e_{SM,ET}\right)$ and 5-d lagged pentad rainfall PP $\left(e_{PP,ET}\right)$ after removing their respective linear regressions with the pentad ET averaged over the 5 d after the initial soil moisture. The partial correlation allows us to examine where the soil moisture–rainfall coupling may be suppressed/enhanced by the process chain through ET. More specifically, we compare the spatial distribution of the partial correlation (ρ(SM,PP)⋅ET) with the spatial distribution of the correlation between initial soil moisture and 5-d lagged pentad rainfall (r(SM,PP). Suppression of the partial correlation relative to the correlation in areas with strong coupling strength supports the role of ET in connecting the initial soil moisture with the 5-d lagged pentad rainfall. Analysis of the two-legged coupling strength and analysis of partial correlation provide a complementary view of the propagation/interruption of different feedbacks through the soil moisture–precipitation coupling process chain (40).

The coupling strengths among soil moisture, ET, and rainfall associated with the MCS/non-MCS components are calculated by replacing each component in Eqs. **1**–**4** with the specific component sourced from MCS/non-MCS rain, to indicate the strengths of their linkages and thus the potential predictability of July rainfall in association with early warm-season rainfall.

For the two-legged coupling indices, their significances are indicated by the correlations between the two variables with *P* values <0.05 (stippled in the *Top* in Figs. 3 and 4 *A*, *C*, and *E*). Their field significances are also tested against the null distribution generated by randomizing the time series of one variable in each leg and recalculating the correlations 100 times (*SI Appendix*, *Supplementary Information Text* and Fig. S11). To reveal areas significantly influenced by the MCS and non-MCS component, we also compare the differences in coupling strengths between the MCS and non-MCS contributed components. This is achieved by computing the coupling indices associated with the MCS and non-MCS components from daily data (instead of pentads) in July of each year and testing the significance of their coupling strength difference using the 22-y samples.

We test the significance of the coupling between initial soil moisture and pentad rainfall against the 500 experiments from the analytical model that constitutes a null distribution with land-driven feedbacks removed. Local significance of coupling index is indicated by stippling in Fig. 4, where the coupling index is greater than the 95th-percentile values from the null distribution. The coupling strengths averaged over the central United States and their variations with lead time are also compared with the 5 to 95% ranges of the coupling strengths from the 500 experiments to indicate their significance (blue and green shading in Fig. 6).

## Data Availability

The data used for our analysis derived from the water tracer-enabled Noah-MP simulations can be accessed from NERSC Gateway Portal (https://portal.nersc.gov/project/m1867/CONUS_WT_NoahMP/) (50). The key analysis codes can be accessed from GitHub (https://github.com/huancui/LandAtmo).
